# Supplementary material for: Cholesterol-rich lipid rafts mediate endocytosis as a common pathway for respiratory syncytial virus entry into different host cells
Source: Microbiol Spectr. 2025 Jul 31;13(9):e01192-25. doi: 10.1128/spectrum.01192-25 (PMC12403703; doi:10.1128/spectrum.01192-25)
Supplement: Supplemental figures and videos legends — Fig. S1 to S5 and Legends for video S1 to S4. [file spectrum.01192-25-s0001.pdf]

Supplementary Materials

Cholesterol-Rich Lipid Rafts Mediate Endocytosis as a Common Pathway for Respiratory Syncytial Virus Entry into Different Host Cells

Anqi Zhou[a, b, #], Bao Xue[b, c, d, #], Jiayi Zhong[b], Junjun Liu[b], Ran Peng[a], Fan Wang[a], Yuan Zhou[b], Jielin Tang[a, b, *], Qi Yang[a, b, *], Xinwen Chen[a, b, c, *]

Correspondence to: Jielin Tang (tang_jielin@gzlab.ac.cn), Qi Yang (yang_qi@gzlab.ac.cn), Xinwen Chen (chen_xinwen@gzlab.ac.cn)

This file includes:

Supplementary figures 1-5

Supplementary videos 1-4

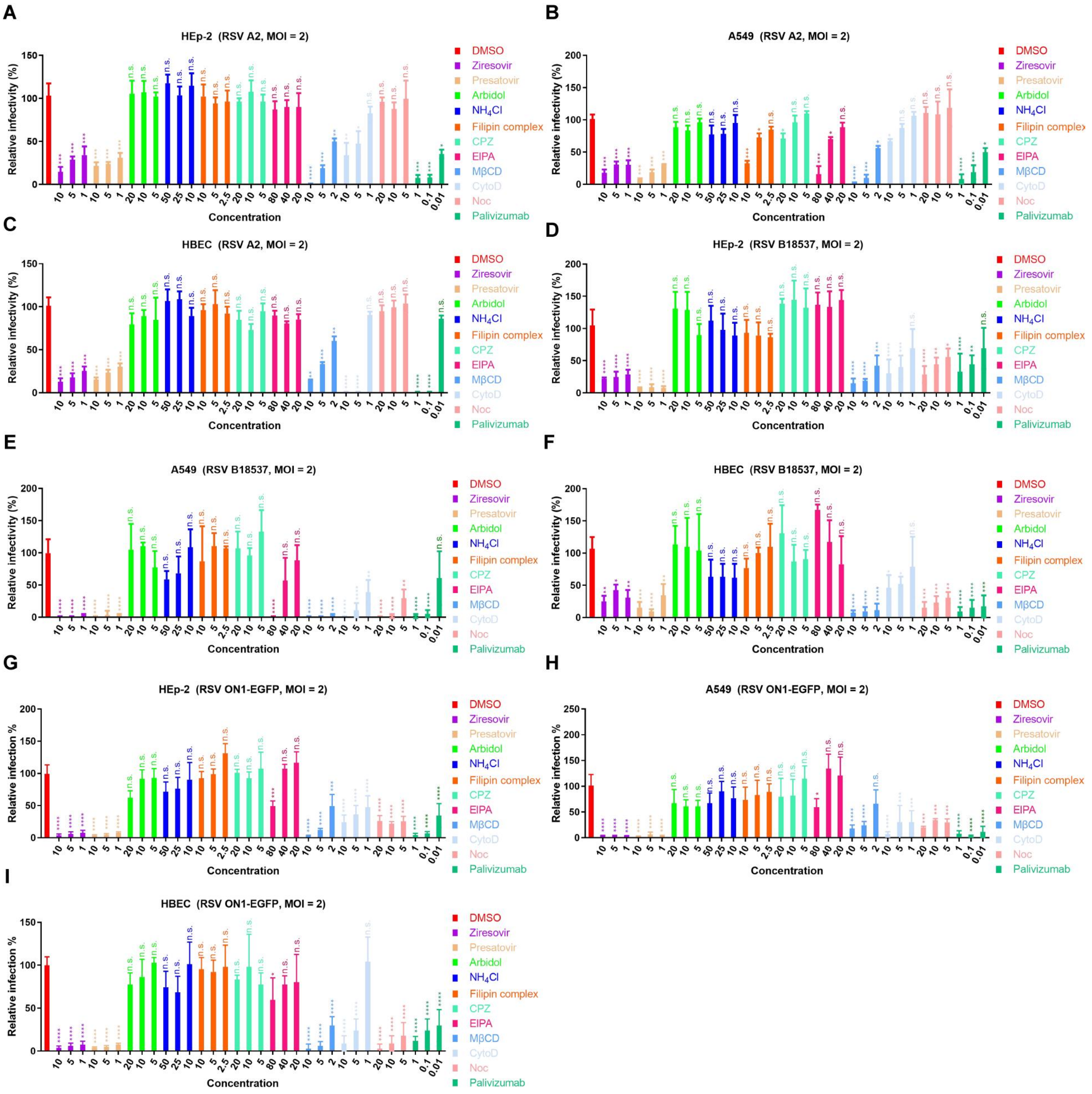

Supplementary Figure 1. RSV entry into host cells is primarily dependent on cholesterol-rich lipid raft-mediated endocytosis. (**A to I**) HEp-2, A549, or HBEC cells were pretreated with ziresovir (1, 5, 10 μM), presatovir (1, 5, 10 μM), arbidol (5, 10, 20 μM), NH_4Cl (10, 25, 50 mM), filipin complex (2.5, 5, 10 μM), CPZ (5, 10, 20 μM), EIPA (20, 40, 80 μM), MβCD (2, 5, 10 mM), Cyto D (1, 5, 10 μM), Noc (5, 10, 20 μM), or palivizumab (0.01, 0.1, 1 μM) for 1 hour. Cells were then infected with either the A2, B18537, or ON1

RSV strains (MOI = 2). After the infection, cells were incubated in the presence of the original inhibitor for a duration of 2 hours. Control cells received an identical dose of dimethyl sulfoxide (DMSO) at the same time points. After the culture media were replaced with those without the inhibitor or the control agent, the cultures were incubated another 22 hours and infection rate was quantified by conducting fluorescence focus assays (FFA). Data are representative of three independent experiments and are presented as mean ± SD. Statistical differences were determined using one-way ANOVA in **A to I**. $*P < 0.05$, $**P < 0.01$, $***P < 0.001$, $****P < 0.0001$; n.s., not significant.

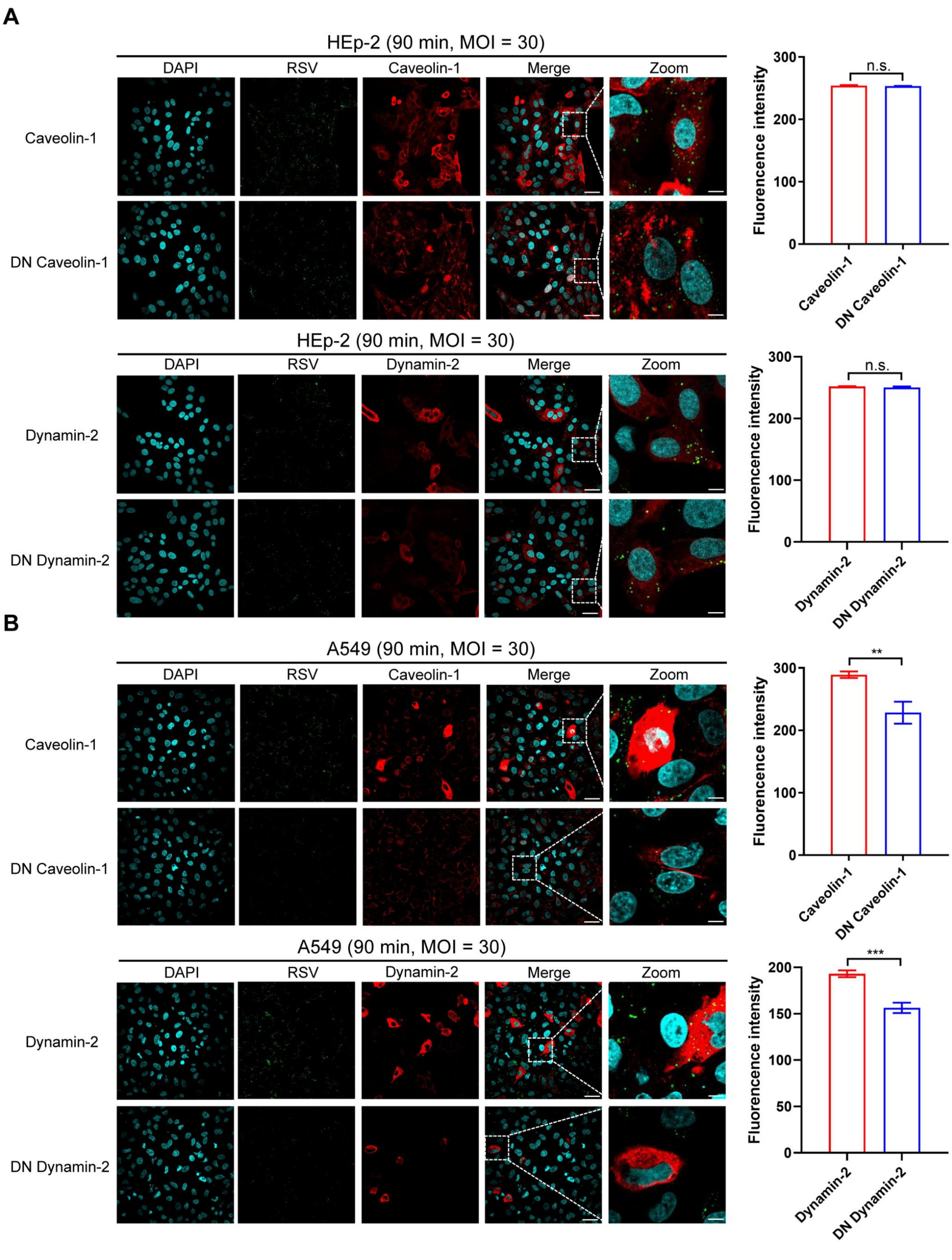

Supplementary Figure 2. RSV entry is rarely dependent on caveolin- and dynamin-mediated endocytosis pathway. (**A, B**) HEp-2 and A549 cells were infected with

RSV (MOI = 30) (90 min, 37°C) and fixed (4% PFA, 15 min, room temperature). Cell nucleus was labeled with DAPI (cyan). Immunodetection of anti-RSV F (green), anti-caveolin-1 and anti-dyanmin-2 (red) antibodies were performed using confocal microscopy. Fluorescence intensity was quantified using ImageJ. Relative quantification was performed by normalizing the data to a control group. n=3 biological replicates. The error bars indicate mean ± SD. Scale bar = 50 or 10 μm.

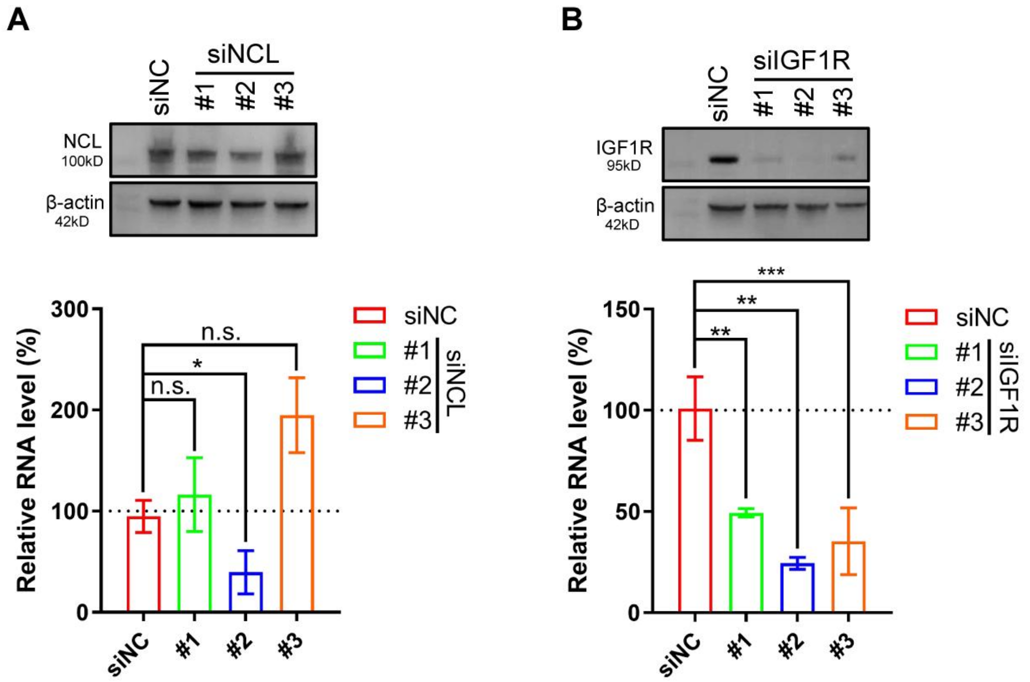

Supplementary Figure 3. Validation of receptors knockdown efficiency. (A, B) HEp-2 cells were transfected with siRNA-NC (control) or siRNAs targeting NCL and IGF1R for 48 hours. Determine the protein expression levels of the NCL and IGF1R by WB, and the relative quantity of mRNAs for NCL and IGF1R was determined by qRT-PCR. n=3 biological replicates. The error bars indicate mean ± SD. Data are representative of three independent experiments and are presented as mean ± SD. Statistical differences were determined by one-way ANOVA. *$P < 0.05$ **$P < 0.01$, ***$P < 0.001$; n.s., not significant.

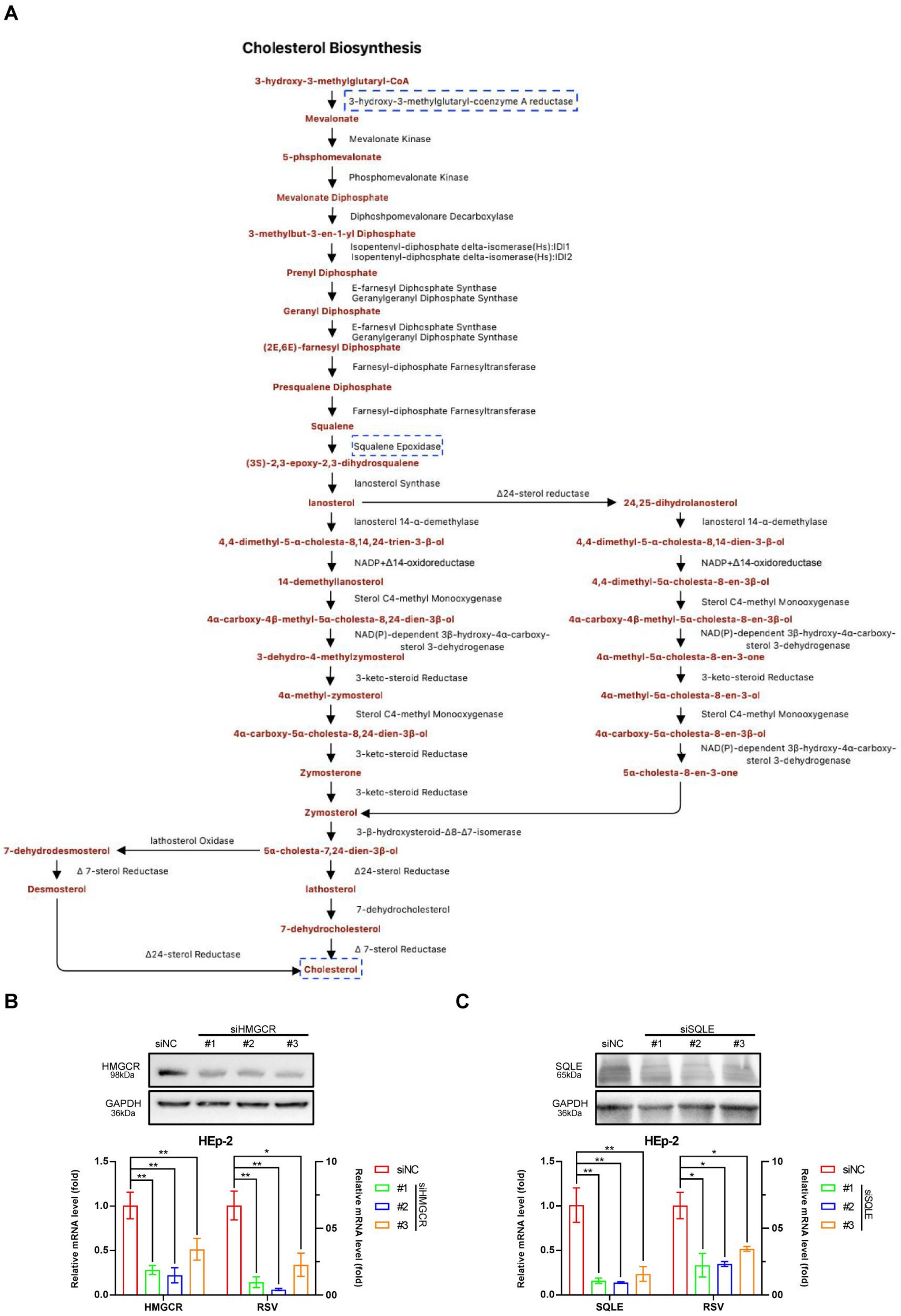
A
Cholesterol Biosynthesis
3-hydroxy-3-methylglutaryl-CoA
3-hydroxy-3-methylglutaryl-coenzyme A reductase
Mevalonate
Mevalonate Kinase
5-phsphomevalonate
Phosphomevalonate Kinase
Mevalonate Diphosphate
Diphoshpomevalonare Decarboxylase
3-methylbut-3-en-1-yl Diphosphate
Isopentenyl-diphosphate delta-isomerase(Hs):IDI1
Isopentenyl-diphosphate delta-isomerase(Hs):IDI2
Prenyl Diphosphate
E-farnesyl Diphosphate Synthase
Geranylgeranyl Diphosphate Synthase
Geranyl Diphosphate
E-farnesyl Diphosphate Synthase
Geranylgeranyl Diphosphate Synthase
(2E,6E)-farnesyl Diphosphate
Farnesyl-diphosphate Farnesyltransferase
Presqualene Diphosphate
Farnesyl-diphosphate Farnesyltransferase
Squalene
Squalene Epoxidase
(3S)-2,3-epoxy-2,3-dihydrosqualene
lanosterol Synthase
lanosterol
Δ24-sterol reductase
24,25-dihydrolanosterol
lanosterol 14-α-demethylase
4,4-dimethyl-5-α-cholesta-8,14,24-trien-3-β-ol
NADP+Δ14-oxidoreductase
14-demethyllanosterol
Sterol C4-methyl Monooxygenase
4α-carboxy-4β-methyl-5α-cholesta-8,24-dien-3β-ol
NAD(P)-dependent 3β-hydroxy-4α-carboxy-sterol 3-dehydrogenase
3-dehydro-4-methylzymosterol
3-keto-steroid Reductase
4α-methyl-zymosterol
Sterol C4-methyl Monooxygenase
4α-carboxy-5α-cholesta-8,24-dien-3β-ol
3-keto-steroid Reductase
Zymosterone
3-keto-steroid Reductase
lanosterol 14-α-demethylase
4,4-dimethyl-5-α-cholesta-8,14-dien-3-β-ol
NADP+Δ14-oxidoreductase
4,4-dimethyl-5α-cholesta-8-en-3β-ol
Sterol C4-methyl Monooxygenase
4α-carboxy-4β-methyl-5α-cholesta-8-en-3β-ol
NAD(P)-dependent 3β-hydroxy-4α-carboxy-sterol 3-dehydrogenase
4α-methyl-5α-cholesta-8-en-3-one
3-keto-steroid Reductase
4α-methyl-5α-cholesta-8-en-3-ol
Sterol C4-methyl Monooxygenase
4α-carboxy-5α-cholesta-8-en-3β-ol
NAD(P)-dependent 3β-hydroxy-4α-carboxy-sterol 3-dehydrogenase
5α-cholesta-8-en-3-one
Zymosterol
3-β-hydroxysteroid-Δ8-Δ7-isomerase
lathosterol Oxidase
7-dehydrodesmosterol
5α-cholesta-7,24-dien-3β-ol
Δ 7-sterol Reductase
Δ24-sterol Reductase
Desmosterol
lathosterol
7-dehydrocholesterol
7-dehydrocholesterol
Δ 7-sterol Reductase
Δ24-sterol Reductase
Cholesterol
B
siHMGCR
siNC
#1
#2
#3
HMGCR
98kDa
GAPDH
36kDa
HEp-2
Relative mRNA level (fold)
1.5
1.0
0.5
0.0
10
05
00
**
HMGCR
RSV
*
siNC
#1
#2
#3
siHMGCR
C
siSQLE
SQLE
65kDa
GAPDH
36kDa
HEp-2
SQLE
RSV
siSQLE

Supplementary Figure 4. Cholesterol biosynthesis pathway and validation of HMGCR and SQLE knockdown efficiency. (**A**) Cholesterol biosynthesis pathway. (**B**) Validation of HMGCE knockdown efficiency. HEp-2 cells were transfected with siRNA-NC (control) or siHMGCR. Determine the protein expression levels of the HMGCR by WB, and the relative quantity of mRNAs for RSV was determined by qRT-PCR. Data are representative of three independent experiments and are presented as mean ± SD. Statistical differences were determined by Student's *t*-test in **B**. (**C**) Validation of SQLE knockdown efficiency. HEp-2 cells were transfected with siRNA-NC (control) or siSQLE. Determine the protein expression levels of the SQLE by WB, and the relative quantity of mRNAs for RSV was determined by qRT-PCR. Data are representative of three independent experiments and are presented as mean ± SD. Statistical differences were determined by Student's *t*-test in **C**. *$P < 0.05$, **$P < 0.01$.

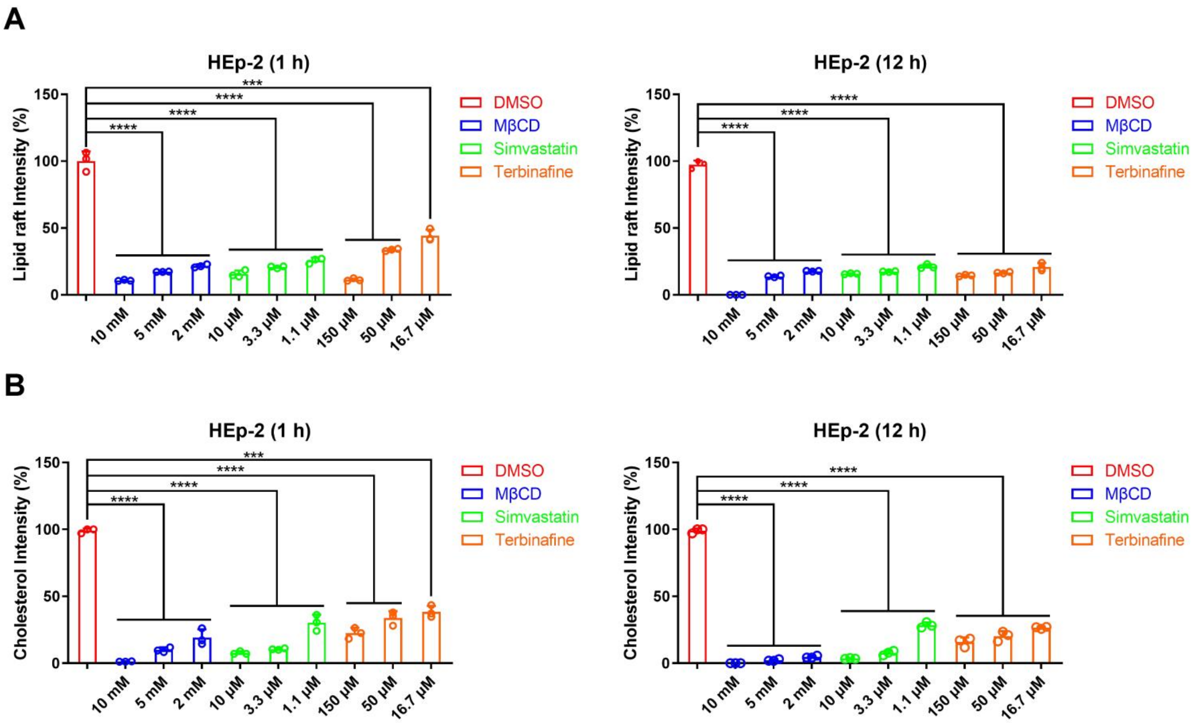

Supplementary Figure 5. The depletion and disruption of cholesterol and lipid rafts by inhibitors. (**A, B**) The inhibitors were treated to HEp-2 cells for 1 hour and 12 hours (37°C) according to the indicated concentrations of MβCD (2, 5, 10 mM), simvastatin (1.1, 3.3, 10 μM), and terbinafine (16.7, 50, 150 μM), respectively, and the control group was treated without the addition of the inhibitors. Lipid rafts were labeled with Alexa 647-CTB, and cholesterol with NBD-cholesterol. The images were photographed using confocal microscopy. Fluorescence intensity was quantified using ImageJ. Relative quantification was performed by normalizing the data to a control group. n=3 biological replicates. The Data are representative of three independent experiments and are presented as mean ± SD. Statistical differences were determined by one-way ANOVA. ***$P < 0.001$, ****$P < 0.0001$.

Supplementary Video 1. HEp-2 cells lipid rafts colocalizes with DiD-labelled RSV particles during viral entry.

Supplementary Video 2. HEp-2 cells cholesterol colocalizes with DiD-labelled RSV particles during viral entry.

Supplementary Video 3. A549 cells lipid rafts colocalizes with DiD-labelled RSV particles during viral entry.

Supplementary Video 4. A549 cells cholesterol colocalizes with DiD-labelled RSV particles during viral entry.
